# Essential Limitations of the Standard THz TDS Method for Substance Detection and Identification and a Way of Overcoming Them

**DOI:** 10.3390/s16040502

**Published:** 2016-04-08

**Authors:** Vyacheslav A. Trofimov, Svetlana A. Varentsova

**Affiliations:** Faculty of Computational Mathematics and Cybernetics, Lomonosov Moscow State University, Leninskiye Gory, Moscow 119992, Russia; svarentsova@gmail.com

**Keywords:** transmitted or reflected THz pulse with a few cycles, pulsed Time-Domain Spectroscopy, spectral dynamics analysis method, detection and identification of substances, integral correlation criteria

## Abstract

Low efficiency of the standard THz TDS method of the detection and identification of substances based on a comparison of the spectrum for the signal under investigation with a standard signal spectrum is demonstrated using the physical experiments conducted under real conditions with a thick paper bag as well as with Si-based semiconductors under laboratory conditions. In fact, standard THz spectroscopy leads to false detection of hazardous substances in neutral samples, which do not contain them. This disadvantage of the THz TDS method can be overcome by using time-dependent THz pulse spectrum analysis. For a quality assessment of the standard substance spectral features presence in the signal under analysis, one may use time-dependent integral correlation criteria.

## 1. Introduction

As it is well-known, a detection and identification of explosives, drugs and other hazardous chemical and biological agents is an urgent security problem. For this problem solving, THz radiation has been widely used during the past twenty years [1,2,3,4,5,6,7,8,9,10,11,12,13,14]. Usually, the detection and identification of substances is based on a comparison of the absorption frequencies of the substance under consideration with the absorption frequencies of a standard substance from a database [5,6,7,8,9,10].

We will call this method the standard THz TDS method. It should be stressed that by standard THz TDS method we do not refer to the way for obtaining and registering the THz signal, but rather to the analysis of the spectral characteristics (Fourier spectra, absorbance or reflectance spectra) of the THz signal reflected from or transmitted through the object. Obviously, this method has well-known disadvantages. For example, many explosives have simulants—ordinary substances with similar absorption frequencies—which makes use of the TDS method not effective enough. Opaque packaging, inhomogeneity of the substance surface, high atmospheric humidity during the measurements also lead to low efficiency of this method [11,12,13,14]. We notice that in [10] the author reported the same disadvantages of THz spectroscopy.

In the present paper, we show the principal limitations of the standard THz TDS method for the detection and identification of substances under real conditions—at long distances of about 3.5 m under room conditions with a relative humidity of about 50%—which differ from the disadvantages mentioned above. For this purpose, we conducted a physical experiment with a neutral substance—a thick paper bag. Another example is the identification of Si-based semiconductors under laboratory conditions—at a short distance of about 30 cm and in dry air with a relative humidity less than 2%. We show the standard THz TDS method determines the spectral features of hazardous substances in the neutral substances in both physical experiment cases. This property is not inherent to any particular installation (for example, to that in the Lomonosov Moscow State University), because all our data were obtained from different laboratories. This fact proves the generality of the phenomenon and results in difficulty for construction a device based on the use of the standard THz TDS method, which can really detect dangerous substances in real conditions, as it will lead to a large number of false positives. A few words about why we chose to investigate semiconductors: currently, almost everyone has in their pocket or purse a cell phone or tablet PC that contains semiconductors. If false detection of explosives instead of cell phones occurs many times, the remote screening efficiency will tend to zero.

To overcome these negative features of THz TDS, we have developed a novel method for the detection and identification of substances by using the broadband THz signal with a few cycles. Its main feature consists in the spectral dynamics analysis of the substance response to THz radiation. We call it the SDA-method.

Earlier, the SDA-method was successfully applied in the transmission mode for the identification of various neutral and dangerous substances [15,16,17]. Later we showed the possibility of applying the SDA-method for this purpose in reflection mode [18]. In [19,20] the integral correlation and likeness criteria of probability assessment for the detection and identification of substance were proposed. In [21,22,23,24,25] the spectral properties of THz pulses measured at long distances of about 3.5 m were investigated by means of proposed modified integral criteria.

As our practice shows, to detect the presence of an explosive in a sample is a simpler task than proving its absence. Let us note that we have demonstrated the presence of explosives in the samples by means of the SDA-method and integral correlation criteria in a number of our previous papers, for example, in [15,20,24,25,26]. In particular, in [26] we showed the possibility to detect the explosive RDX in the plastic explosive PWM C4 with an inhomogeneous (rough and concave) surface in reflection mode using the integral correlation criteria (ICC).

By analogy with the “electronic nose”, the method discussed in [19,20,21,22,23,24,25,26] was called by us a “terahertz nose”. We recall that the “electronic nose” [27,28,29] is a multi-sensor system for the detection and analysis of multi-component gas mixtures. Identification in the modern sensor system occurs on the basis of chemical or physical sensor properties’ changes (e.g., a change in conductivity, the change of mass, fluorescence, *etc.*). Disadvantages of this technology are well-known and are associated with the identification of hidden objects, for example, if packed in polyethylene or paper, or hidden under clothes.

We emphasize that the SDA-method is based on processing of the reflected or transmitted THz signal. The method does not depend on the particular installation which was used for the THz signal measurement. In our opinion, it is a big advantage of our method and that is why we use in our article THz signals obtained in different laboratories. Let us stress once again, that the main aim of this paper is to demonstrate the principal limitations of the standard THz TDS method for the detection and identification of substances.

It should also be noted that besides the security screening applications, the demonstrated ultrafast THz spectroscopy approach also provides new opportunities to study technologically-related materials ranging from nanostructures to strongly correlated electron materials [30,31].

The measurements of the THz signal from thick paper bag at long distance were made in Lomonosov Moscow State University (Moscow, Russia). THz signals transmitted through RDX were registered in the Center for Terahertz Research, Rensselaer Polytechnic Institute (New York, NY, USA) and were provided to us by Xi-Cheng Zhang. The examples with the thick bag and semiconductors were previously partially discussed in [24,25].

## 2. Description of the Setup and Measurements

In the physical experiment with a thick paper bag, we exploit an EKSPLA commercially available THz spectrometer developed by Teravil Inc. (Vilnius, Lithuania). It is made up of a femtosecond fiber laser, which generates laser pulses with an average power 1 W at 1030 nm centre wavelength, with 75 MHz repetition rate, and its pulse duration is equal to 80 fs. Low-temperature grown GaBiAs is used as photoconductor. The spectral range of the spectrometer is 0.1–5.0 THz, signal-to-noise is better than 10^3^:1 (at 2 THz), 10^5^:1 (at 1 THz) and 10^6^:1 (at 0.4 THz). We use a parabolic mirror for the THz beam focusing on the object under investigation. Because the femtosecond fiber laser has low average power and the laser beam splits many times, we use additional flat mirror behind the object. Therefore, our setup operates in reflection-transmission mode simultaneously for each measurement.

**Figure 1:**
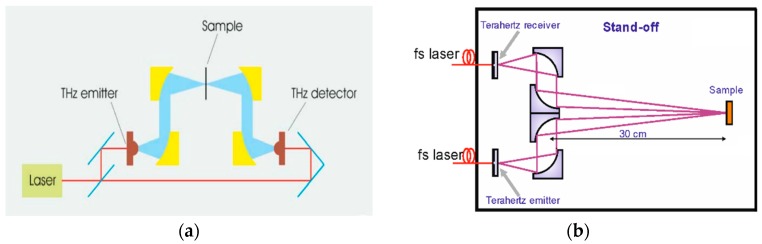
The principal scheme of the system for the transmission (**a**) and reflection (**b**) mode.

The distance between the parabolic mirror and the object was about to 3.5 m. The principal scheme of the system is shown in Figure 1 for the transmission and reflection mode. A photo of the experimental setup for measurements at long distance can be found in [26].

A THz signal transmitted through the explosive RDX in transmission mode was measured using a TPS 3000 unit (Teraview, Cambridge, UK) in the standard configuration [26]. The main parameters of the system are: spectral range 0.06–3.6 THz, signal-to-noise better than 4000:1, dynamic range higher than 3 OD in the range 2 to 100 cm^−1^, spectral resolution 0.06 THz and rapid scan mode with 30 scans/s. Measurements were made at the distance of not more than 30 cm between a sample and the mirrors, as described in detail in [9]; the temperature in both cases was 22 °C and the only difference is that in our case the RDX signal was measured in air with a relative humidity of about 50%.

## 3. Spectral Analysis

### 3.1. Spectral Properties of the Thick Bag Sample

**Figure 2:**
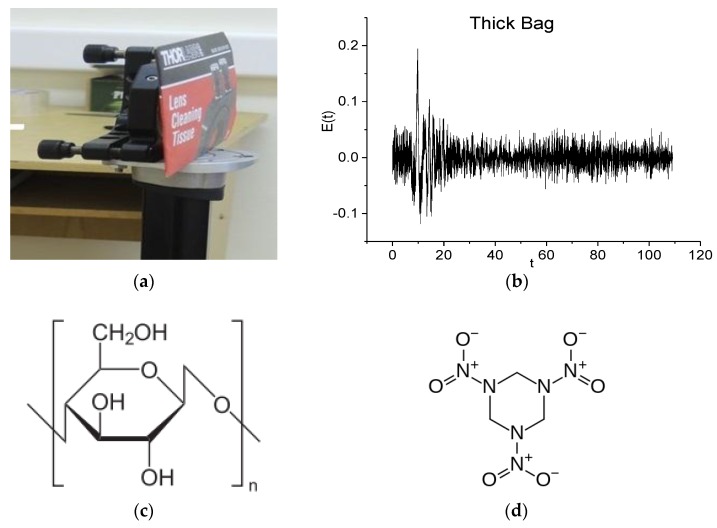
Thick paper bag (**a**) investigated in real conditions at long distance of about 3.5 m; the Thick Bag signal (**b**) at the time interval *t* = [0, 110] ps; chemical formulae of cellulose (**c**) and RDX (**d**).

In Figure 2a, we present the sample investigated under real conditions at a long distance of about 3.5 m; it is a thick paper bag with a thickness of 5–7 mm. The measurement was carried out at a temperature of 18 °C and relative humidity of about 50%. We shall call this THz signal the Thick Bag signal.

Figure 2b shows the Thick Bag signal registered at the time interval *t* = [0, 110] ps. The pronounced sub-pulses, which are typical for reflected THz signals and caused by multiple reflections from the inner boundaries of the sample, are absent in (b). At the same time, high noise is observed in the signal. It should be stressed that the thick paper sample consists essentially of cellulose. In Figure 2c the chemical formula of cellulose is presented [32]. Note that reference [33] reports the absorption frequencies ν = 2.15 THz and ν = 3.03 THz for pure microcrystalline cellulose samples measured under laboratory conditions. In Figure 2d we show the formula of the explosive RDX [34], as we compare below the spectral properties of the thick paper bag and RDX.

The commonly used TDS deals with the spectrum of the main pulse of a THs signal transmitted or reflected from the object. As one can see from Figure 2b, the main pulse is observed in the time interval *t* = [0, 25] ps duration *t* = 25 ps. In order to get its spectrum and to avoid the influence of noise we cut the main pulse out from the long THz signal measured in this time interval.

**Figure 3:**
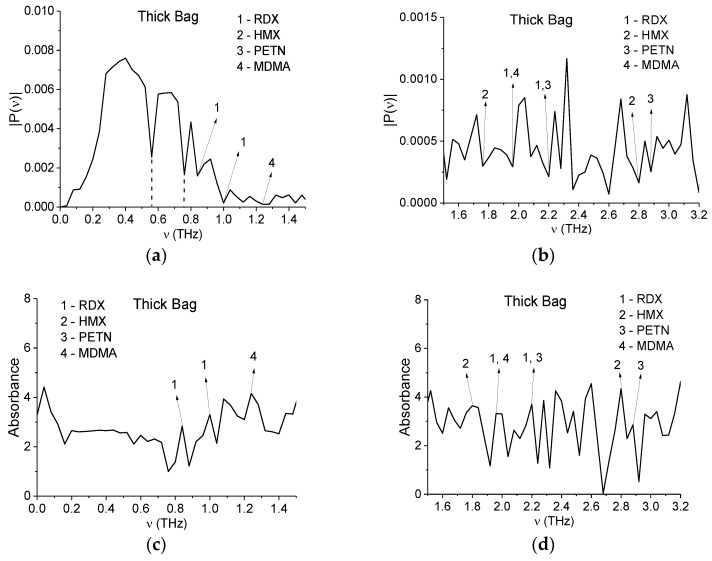
Fourier spectrum of the main pulse of the Thick Bag signal (**a**,**b**) and the absorbance of the sample (**c**,**d**) in the frequency ranges ν = [0, 1.5] THz (**a**,**c**); ν = [1.5, 3.2] THz (**b**,**d**) calculated with frequency resolution Δν = 0.04 THz.

Figure 3 shows the Fourier spectrum of the main pulse for the Thick Bag signal in the frequency ranges ν = [0, 1.5] THz (a) and ν = [1.5, 3.2] THz (b) calculated in the time interval *t* = [0, 25] ps with frequency resolution Δν = 0.04 THz. In Figure 3c,d the sample absorbance *A* is calculated in the same frequency ranges. Here:

(1) $$A=-\log_{10}(|P(\nu )|/|P_{REF}(\nu )|)$$

where |P(ν)|, $\left|P_{REF}(\nu )\right|$ are the absolute spectral amplitude values of the measured and reference signals, respectively [35]. The Fourier spectrum of the whole signal can be found in [24]. In [24] we showed that the minima corresponding to frequencies ν = 0.56, 0.76 THz in Figure 3a may be caused by water vapor contained in the air and the superposition of reflected and transmitted signals. The absence of absorbance maxima at these frequencies in Figure 3c confirms our conclusion.

It is obvious that hazardous substances are absent in the thick paper bag sample. However, in the Fourier spectrum and absorbance of the signal measured under real conditions, it is possible to observe the spectral features of many dangerous substances. According to [6,7,9], the characteristic absorption frequencies of the explosives RDX, HMX, and PETN are: ν = 0.82, 1.05, 1.36, 1.54, 1.95, 2.19, 3.0 THz for RDX; ν = 1.78, 2.51, 2.82 THz for HMX; ν = 2.0, 2.16, 2.84 THz for PETN. In Figure 3, one can see minima in Figure 3a,b and maxima in Figure 3c,d, corresponding to the absorption frequencies of the explosives RDX, HMX, PETN and the illicit drug MDMA [24].

Some of these frequencies ν = 2.16, 2.88 THz are close to those of cellulose ν = 2.15 THz and ν = 3.03 THz [33], the others may be influenced by agents used during the manufacture and preserved in the samples.

This example shows that the main problem of identifying substances with the standard THz TDS method is not that the method determines the frequencies of these substance simulants, but in that the method is not able to show the absence of hazardous substances in the sample.

### 3.2. Spectral Properties of Si-Based Semiconductors

In the next example, we show that the application of the standard THz TDS method for the identification of substances can lead to inaccurate results even in the case of measurements under laboratory conditions—over a short distance of 30–40 cm, and at room temperature and low humidity.

Below we investigated the spectral properties of n-Si, p-Si semiconductors and silicon wafer with resistivity 40 Om·cm and measured with 12 mm aperture, in comparison with spectral properties of dangerous substances. We will call these THz signals n-Si, p-Si and Si-40-12 for brevity. Measurements of n-Si, p-Si signals were performed at Capital Normal University (Beijing, China) and of Si-40-12 signal at South China Normal University (Guangzhou, China).

The measurements of n-Si, p-Si signals were performed using a Ti:sapphire regenerative amplifier delivering ultrashort optical pulses with a duration of 100 fs and a central wavelength of 800 nm at a repetition rate of 1 kHz. The output of the laser was divided by beam splitters into three portions: a terahertz generation beam, probe beam, and pump beam. The generation pulse was incident on a 2-mm-thick (110) ZnTe emitter to generate the terahertz pulse via optical rectification [36]. The terahertz wave was normally incident to the sample. The transmitted THz signal was detected by free-space electro-optic sampling in a 1-mm-thick <110> ZnTe crystal with the sampling pulse [37]. Then the signal was collected by a lock-in amplifier with its frequency locked to an optical chopper. The system was purged by nitrogen to prevent absorptions by atmospheric humidity. The experiments were performed at room temperature.

**Figure 4:**
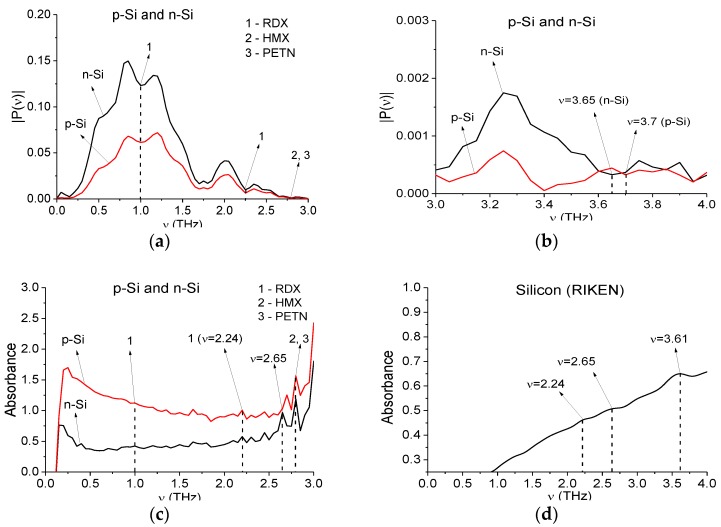
Fourier spectrum (**a**,**b**) and absorbance (**c**) of the n-Si and p-Si THz signals in the frequency ranges ν = [0.0, 3.0] (**a**,**с**); [3.0, 4.0] THz (**b**); absorbance of monocrystalline silicon in the frequency range ν = [0.0, 3.0] THz (**d**).

Figure 4 shows the Fourier spectra of the main pulses of the n-Si and p-Si signals in the frequency range ν = [0.0, 3.0] THz (a) and ν = [3.0, 4.0] THz (b), in Figure 4c—absorbance in the frequency range ν = [0.0, 3.0] THz. One can see minima in Figure 4a and maxima in Figure 4c corresponding to the absorption frequencies of the explosives RDX, HMX, PETN [9].

In Figure 4d the absorbance of pure monocrystalline silicon is shown, using data from a THz database [38] (Teraphotonics Laboratory, RIKEN, Sendai, Japan). The paper [39] reported that the frequency ν = 3.6 THz is the well-resolved absorption feature of high-resistivity, float-zone silicon. Note that in (d) one can see maxima at frequencies ν = 2.24, 2.65, 3.61 THz. The first one is the same as the extremes in (a,c), marked by “1”. The second maximum in (d) coincides with the maximum n-Si absorbance at ν = 2.65 THz (c). For the p-Si signal the corresponding maximum in (c) is shifted to ν = 2.7 THz. The third maximum in (d) at a frequency ν = 3.61 THz is close to the minimum of the n-Si signal at frequency ν = 3.65 THz in (b) and close to the pure monocrystalline silicon absorption frequency ν = 3.6 THz [39]. At the same time, for the p-Si signal the corresponding minimum in (b) is shifted to ν = 3.7 THz. That is, the semiconductor samples n-Si and p-Si demonstrate spectral features, which are close to those of both of pure silicon and explosives.

The Si-40-12 signal was measured also in transmission mode, at a short distance of about 30–40 cm from the receiver at room temperature in the open air with non-zero humidity using the standard THz TDS unit. The measurement procedure is similar to that described in [40].

**Figure 5:**
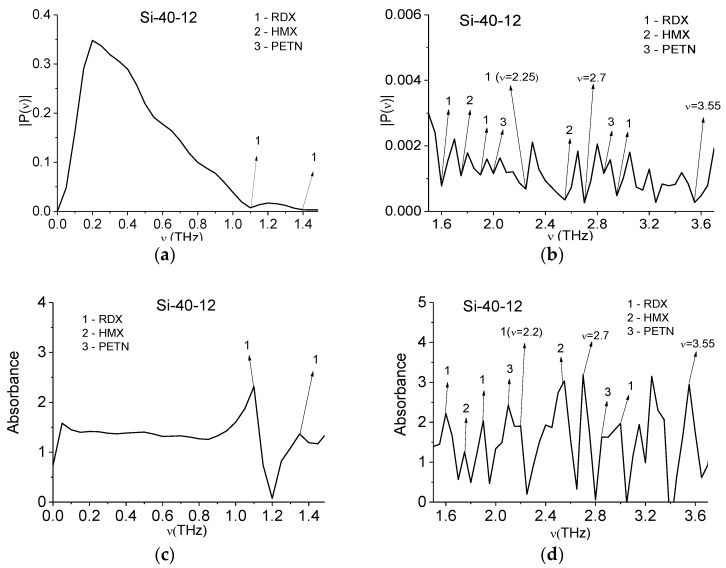
Fourier spectrum (**a**,**b**) and absorbance (**c**,**d**) of the main pulse of the Si-40-12 signal in the frequency ranges ν = [0, 1.6] THz (**a**,**c**); [1.5, 3.2] THz (**b**,**d**).

In Figure 5 the Fourier spectrum (a,b) and absorbance (c,d) of the main pulse of the Si-40-12 signal is presented in the frequency ranges ν = [0, 1.5] THz (a,c); [1.5, 3.7] THz (b,d). The Fourier spectrum and absorbance of the main pulse were calculated in the time interval *t* = [4, 24] ps length *T* = 20 ps. Note that in (b) one can see minima at frequencies ν = 2.25, 2.7, 3.55 THz, which are close to the absorption frequencies of pure silicon ν = 2.24, 2.65, 3.61 THz [38], ν = 3.6 THz [39]. The corresponding maxima in (d) are ν = 2.2, 2.7, 3.55 THz. That is, the silicon-based semiconductor samples from different laboratories (n-Si, p-Si and Si-40-12) demonstrate spectral features which are close to those of pure silicon.

One can see in Figure 5a,b minima and in Figure 5c,d maxima corresponding to the absorption frequencies of the explosives RDX, HMX and PETN. Note that in Figure 5 in the spectrum and absorbance there are more extremes that in Figure 4. This may be caused by the influence of the environment, including water vapor in the air. As in the previous case with n-Si and p-Si semiconductors, the Si-40-12 sample also demonstrates spectral features of explosives.

Therefore, the standard THz TDS method has fundamental limitations which do not allow its use for the detection and identification of substances under real conditions. However, integral correlation criteria together with the SDA-method allow one to show the absence of explosives in the samples of a thick paper bag and Si-based semiconductors.

## 4. SDA-Method and Modified Integral Correlation Criteria

### 4.1. Advantages of the Spectral Dynamics Analysis Method (SDA-Method)

It is obvious that the standard THz TDS method does not take into account the changes in instantaneous spectral intensities; it gives information about the spectrum averaged over the time of pulse registration. At the same time, the response of the medium to the action of the THz pulse with a few cycles is essentially non-stationary. The analysis of the evolution of the spectral intensities in time (spectral dynamics) at the chosen frequency ν allows one to get much more information about the substance than the analysis of the spectrum alone.

For the transmitted THz signals obtained under laboratory conditions, it is often sufficient to use the TDS method for identifying substances. However, in actual use the TDS method is inefficient because the THz signals detected under real conditions often have noisy Fourier spectra, which may be distorted by water vapor, the influence of the packaging material, and so on. In this case, it becomes necessary to develop new criteria that are not based on a comparison of the absorption coefficients for evaluating the detection and identification of substances. These effective criteria can be integral correlation and likeness criteria proposed by us on the basis of the application of SDA-method [19,20].

In order to get information about the characteristic absorption frequencies and relaxation times, we analyze the dynamics of the spectral intensities of the signal under investigation. For this purpose, we move the spectral dynamics of standard THz signal at characteristic absorption frequency along the spectral dynamics of the investigated signal in the chosen time intervals. As a standard signal, we use a THz signal transmitted through the sample with the desired substance and measured in laboratory conditions, or a signal measured in ambient air in view of water absorption frequencies. Analyzing the integral correlation between these spectral dynamics, we can conclude about the presence or absence of characteristic features of the standard substance in the sample.

Note that the frequency resolution is obviously dependent on the length of time interval *T* for which the measurement are performed. As a rule, the duration *T* was approximately 100 ps. Consequently, the minimum frequency difference Δν, which can be resolved by computer processing, is 10 GHz.

### 4.2. SDA-Method

In Section 4.2 and Section 4.3 we recall the basic steps in constructing the spectral line dynamics in the SDA-method and in calculating the integral correlation criteria, see for example, [23,24,25,26].

The proposed integral criteria are based on the analysis of total correlation characteristics over relatively short time intervals and taking into account the spectral brightness of frequencies $\nu_{1}$ and $\nu_{2}$ during these time intervals. Here $\nu_{1}$ is a chosen frequency of the signal under investigation and $\nu_{2}$ is a known absorption frequency of the standard signal. The correlation characteristics in turn, are based on dynamics of spectral amplitudes of THz signal under investigation at the chosen frequencies.

Let E(t) be the transmitted or reflected THz signal measured in the time interval [*t_b_*, *t_e_*]. Information about the time evolution of the full spectrum or part of it can be obtained by using a time window with duration (length) *T* that slides along the signal. At each step, the time window is shifted on the chosen time interval Δ, and then the Fourier transform is applied to the function E(t) in this window. To avoid any “spreading” of the spectrum, we multiply the signal E(t) by function *g*(*t*), which tends to zero very quickly at the ends of the window. In order to construct the dynamics of the spectral line (or evolution of the modulus of spectral amplitude) $P(\nu ,t_{j})$ of the function E(t) at chosen frequency ν, the Fourier-Gabor transform is carried out in each time window of length *T*:

(2) $$P(\nu ,t_{j})=\frac{1}{T}\int_{t_{j}}^{t_{j}+T}E(t)\cdot g\left(t\right)e^{-i2\pi \nu (t-t_{j})}dt,\;g(t)=e^{-{\left(\frac{t-t_{c}}{0.5T}\right)}^{k}}$$

where $t_{j}$ is a time of window beginning, *j* is a serial number of window, *T* is its length, ν is a frequency. The units of $t_{j}$, *T*, Δ and ν are ps and THz, respectively. Then we calculate the absolute value of spectral amplitude $P(\nu ,t_{j})$ in each time interval as its value at the end of the window in order to align the beginning of the physical pulse and its representation in the SDA method:

(3) $$\left|P_{\nu }(t_{j})|=|P(\nu ,t_{j}+T)\right|$$

In this paper, we choose parameters of window length, its shift and power *k* in the following way: the window length T = 2.8 ps, the window shift Δ = 0.2 ps and *k* = 20.

### 4.3. Integral Correlation Criteria

In order to investigate the integral correlation between the dynamics of spectral lines for the reflected or transmitted signal S(t) and the standard transmitted signal s(t) at chosen frequencies, we introduce the following notation: we denote the discrete set of absolute values of spectral amplitudes of the standard transmitted signal s(t) at the chosen frequency ν (see Equations (2) and (3)) as $p_{\nu }=\left\{\left|p_{\nu }(t_{m})\right|\right\}$, $m=1,...,M_{1}$. The corresponding set of absolute values of spectral amplitudes of the long reflected (or transmitted) THz signal S(t) at frequency ν is denoted as $P_{\nu }=\left\{\left|P_{\nu }(t_{m})\right|\right\}$, $m=1,...,M_{2}$, and its part with $M_{1}$ components, which begins at point $t_{n}$, as $P_{\nu }^{\left(n\right)}=\left\{\left|P_{\nu }^{\left(n\right)}(t_{n+m})\right|\right\}$. Here $M_{1}$ and $M_{2}$ are the numbers of points in the corresponding dynamics; they depend on the dynamics construction parameters—the window length T and its shift Δ, see Equations (2) and (3). Both sets $p_{\nu }=\left\{\left|p_{\nu }(t_{m})\right|\right\}$ and $P_{\nu }^{\left(n\right)}=\left\{\left|P_{\nu }^{\left(n\right)}(t_{n+m})\right|\right\}$ must be averaged at each step $t_{n}$ to avoid the influence of constant components of sets $p_{\nu }$ and $P_{\nu }^{\left(n\right)}$ on correlation. Moving the set $p_{\nu_{1}}$ along the set $P_{\nu_{2}}$, we get in each point $t_{n}$ the correlation coefficient:

(4) $$c_{p,P}(t_{n})=\sum_{m=0}^{M_{1}-1}\left(|p_{\nu_{1}}(t_{m})|-\bar{p_{\nu_{1}}})\cdot (|P_{\nu_{2}}(t_{m+n})|-\bar{P_{\nu_{2}}}\right)/||p_{\nu_{1}}-\bar{p_{\nu_{1}}}||\cdot ||P_{\nu_{2}}^{\left(n\right)}-\bar{P_{\nu_{2}}}||$$

where $\bar{p_{\nu_{1}}}=\sum_{m=0}^{M_{1}-1}\left|p_{\nu_{1}}(t_{m})\right|/M_{1}$, $\bar{P_{\nu_{2}}}=\sum_{m=0}^{M_{1}-1}\left|P_{\nu_{2}}(t_{m+n})\right|/M_{1}$.

Then using the correlation coefficient $c_{p,P}(t_{n})$ for two spectral dynamics, we consider the following integral characteristic for the detection and identification problem [20]:

(5) $$C_{p,P}(t_{n})=\sum_{m=0}^{n}\left|c_{p,P}(t_{m})\right|,\;n=0,...,M_{2}-M_{1}$$

In the present paper, we use the modified criterion on the base of Equation (5), which takes into account the spectral brightness of each frequency $\nu_{1}$ and $\nu_{2}$ during the interval of correlation:

(6) $$CW_{p,P}(t_{n})=\sum_{m=0}^{n}\left|c_{p,P}(t_{m})\right|w_{1}w_{2},\;n=0,...,M_{2}-M_{1}$$

where $w_{1}=w(|P(\nu_{1})|)$, $w_{2}=w(|P(\nu_{2})|)$ are the weight coefficients during the interval of correlation.

Along with the criterion (5) we deal with another criterion, in which the sets $p_{\nu_{1}}^{2}=\left\{|p_{\nu_{1}}(t_{m}){|}^{2}\right\},\;P_{\nu_{2}}^{2}=\left\{|P_{\nu_{2}}(t_{m}){|}^{2}\right\}$ will be used instead of sets $p_{\nu_{1}}$ and $P_{\nu_{2}}$:

(7) $$CW_{p,P}^{SQ}(t_{n})=\sum_{m=0}^{n}\left|c_{p^{2},P^{2}}(t_{m})\right|{w_{1}}^{2}{w_{2}}^{2},\;n=0,...,M_{2}-M_{1}$$

It should be stressed that if $w_{1}=1$ and $w_{2}=1$, we get integral characteristic Equation (5).

In [20] a criterion was introduced that assesses the similarity (or likeness) of two spectral line dynamics:

(8) $$L_{p,P}(t_{n})=\sum_{m=0}^{n}l_{p,P}(t_{m}),\;n=0,...,M_{2}-M_{1}$$

where:

(9) $$l_{p,P}(t_{n})=1-\frac{\left||{\left(p_{\nu_{1}}-\bar{p_{\nu_{1}}}\right)}_{N}-{\left({P_{\nu_{2}}}^{\left(n\right)}-\bar{P_{\nu_{2}}}\right)}_{N}|\right|}{\left||{\left(p_{\nu_{1}}-\bar{p_{\nu_{1}}}\right)}_{N}||+||{\left({P_{\nu_{2}}}^{\left(n\right)}-\bar{P_{\nu_{2}}}\right)}_{N}|\right|},\;n=0,...,M_{2}-M_{1}$$

The subscript N indicates that corresponding sets in Equation (9) are to be normalized, for example, in $L_{2}$. In this paper, we give an example of application of this criterion for identification.

## 5. Identification Based on Integral Correlation Criteria

### 5.1. Detecting the Absence of RDX in the Thick Bag Sample

**Figure 6:**
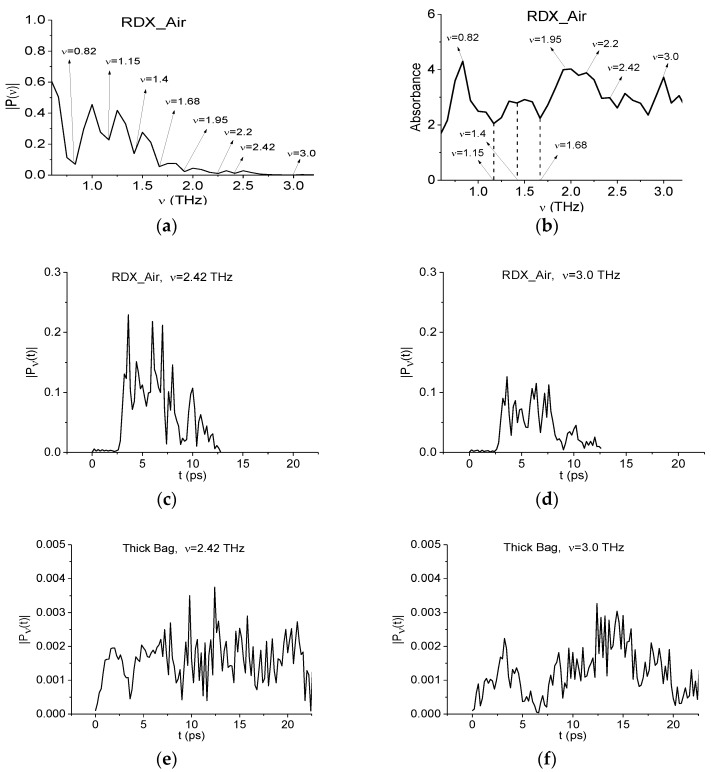
Fourier spectrum of the RDX_Air THz signal (**a**) and absorbance (**b**) in the frequency range ν = [0.6, 3.2] THz; dynamics of spectral lines of the RDX_Air (**c**,**d**) and Thick Bag (**e**,**f**) signals at frequencies ν = 2.42 THz (**c**,**e**); 3.0 THz (**d**,**f**).

In order to show the absence of explosive RDX in the Thick Bag sample and Si-based semiconductors, we will use the THz signal transmitted through the tablet containing 10% RDX and 90% PE in the ambient air as a standard one (RDX_Air signal). Figure 6 shows the Fourier spectrum of the RDX_Air signal Figure 6a and absorbance in the frequency range ν = [0.6, 3.2] THz. One can see in Figure 6a minima at frequencies ν = 1.15, 1.4, 1.68 THz. At the same time, the maxima of absorbance at these frequencies are absent in Figure 6b, *i.e.*, these minima are false absorption frequencies in the Fourier spectrum of the RDX_Air signal. Their appearance is caused by the presence of strong absorption of signal energy by water vapor. However, the minima of the Fourier spectrum of the RDX_Air signal at frequencies ν = 0.82, 1.95, 2.2, 2.42, 3.0 THz (a) coincide with maxima of the absorbance (b), and they can be used to identify the explosive RDX. Note that these frequencies are in a good agreement with the RDX absorption frequencies given in [6,7,9].

In the spectrum of the Thick Bag signal (a) there is no minimum at frequency, which is equal or close to the characteristic minimum of the RDX_Air spectrum at ν = 2.42 THz. In addition the minimum at ν = 3.0 THz is also absent in this spectrum, so in order to show the absence of RDX in the Thick Bag sample with the help of modified criteria (6) and (7), we will use the dynamics of the spectral lines of RDX_Air signal at frequencies ν = 2.42, 3.0 THz as the standard ones. The corresponding spectral lines dynamics $\left|P_{\nu }(t)\right|$ for the RDX_Air signal are depicted in Figure 6c,d, and for the Thick Bag signal—in Figure 6e,f. Note that each dynamics have their own individual shape.

Here and below the frequency ν for the signal under investigation is detected if the corresponding characteristic $CW_{p,P}(t_{n})$ ($C_{p,P}(t_{n})$, $L_{p,P}(t_{n})$ or $CW_{p,P}^{SQ}(t_{n})$) calculated for the pair (ν, ν_1_) lies above all other characteristics in the frequency detection range (FDR). Here ν_1_ is the chosen absorption frequency of the standard signal. As a rule, the boundaries of the FDR are extremes of the spectrum closest to the frequency under investigation. *Vice versa*, the frequency ν is not detected if there is at least one other characteristic lying above it in this range of frequencies.

It should be noted that earlier we investigated the influence of the various time intervals chosen for the calculations of the integral correlation criteria (ICC), on the detection and identification of substances. As usual, the main pulse of a THz signal is used for this purpose in transmission mode. In [21] the ICC were applied for the detection and identification of amphetamine-type stimulants using the main pulse of a transmitted THz signal; the measurements were carried out under laboratory conditions—at the short distance of 30 cm from the receiver and low humidity (less than 2%). In [24] we applied ICC for the investigation of the spectral properties of chocolate under real conditions—at a distance of about 3.5 m and high relative humidity of about 50%. It was shown that detection and identification are possible not only on the time interval containing the main pulse but also on the long time interval which follows it. As for the reflected THz signals, in many cases, the main pulse of the reflected signal does not allow one to carry out the identification. In [26] we showed the possibility to detect plastic explosive PWM C4 with inhomogeneous surface using not only the first sub-pulse following the main pulse of the reflected THz signal but the temporal response of the medium at longer intervals, which do not contain both the main pulse and the first sub-pulse. Since in the present paper we discuss the shortcomings of the standard THz TDS method, here and in the following examples, we apply the ICC on the time intervals, which contain only the main pulses of the measured THz signals.

**Figure 7:**
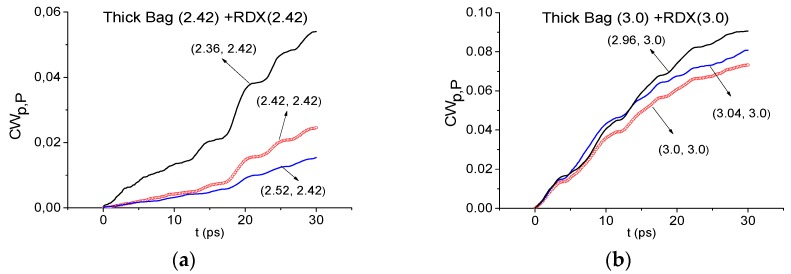
Time-dependent integral characteristic *CW_p,P_*(*t_n_*) calculated for the frequencies ν = 2.42 THz (**a**); 3.0 THz (**b**) if the modified integral criterion is applied for the Thick Bag signal and RDX_Air as a standard signal.

In Figure 7 the integral characteristics *CW_p,P_*(*t_n_*) are shown in the time interval, containing the main pulse of the Thick Bag THz signal, calculated for the frequencies ν = 2.42 THz (a), 3.0 THz (b) for the Thick Bag signal with RDX_Air as a standard one. In both cases these frequencies are not detected in the ranges ν = [2.36, 2.52] THz (a) and ν = [2.96, 3.04] THz (b). Therefore, frequencies ν = 2.42, 3.0 THz (a,b) are not absorption frequencies of RDX in the Thick Bag signal and explosive RDX is absent in the sample with thick paper bag. In the same way, it is possible to show the absence of explosives HMX, PETN and illicit drug MDMA in this sample.

Note that integral characteristics $CW_{p,P}^{SQ}(t_{n})$ give the same result as *CW_p,P_*(*t_n_*) but increase the contrast of detection of the corresponding pairs. They can be used in the case of close or coinciding lines for different pairs of frequencies, when detection by characteristics *CW_p,P_*(*t_n_*) alone is difficult, see [21].

### 5.2. Detecting Paper in the Sample

Below we detect paper in the Thick Bag sample by means of integral correlation Criterias (6) and (7). For this purpose we will use the standard transmitted THz signal Paper_phase(+80.68), which was measured at short distance of about 30 cm from a receiver at room temperature at South China Normal University (Guangzhou, China). The measurement procedure is similar to that described in [40].

**Figure 8:**
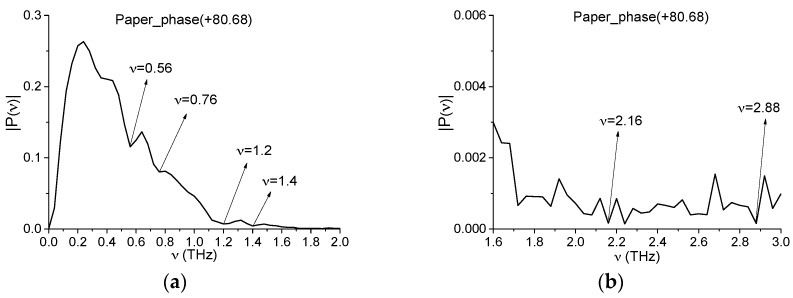
Fourier spectrum of the main pulse of the transmitted Paper_phase(+80.68) THz signal in the frequency ranges ν = [0, 2.0] THz (**a**) and ν = [1.6, 3.0] THz (**b**).

Figure 8 shows the corresponding Fourier spectrum of the main pulse of the transmitted Paper_phase(+80.68) THz signal in the frequency ranges ν = [0, 2.0] THz (a) and ν = [1.6, 3.0] THz (b). Comparing the Fourier spectrum of the standard signal (Figure 8) with the spectrum of the Thick Bag signal (Figure 3), one can see the common or close minima at frequencies ν = 0.56, 0.76, 2.2, 2.88 THz. To find paper in the Thick Bag sample, we will use the spectral lines dynamics of the standard transmitted Paper_phase(+80.68) signal at frequencies ν = 2.16, 2.88 THz which are close to the absorption frequencies of cellulose ν = 2.15, 3.03 THz [29]. The shift of absorption frequencies of the Paper_phase(+80.68) signal may be caused by influence of additional substances in the paper (pigments, fillers, *etc.*).

**Figure 9:**
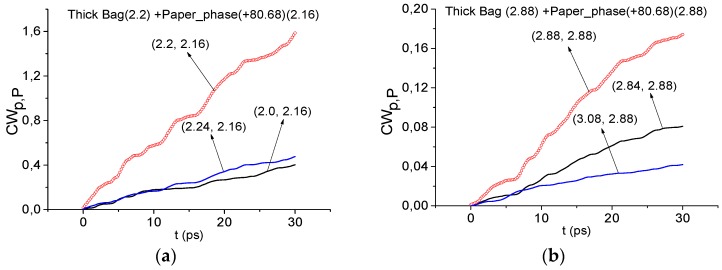
Time-dependent integral characteristics *CW_p,P_*(*t_n_*) detection frequencies ν = 2.2 THz (**a**); 2.88 THz (**b**) for the Thick Bag signal and Paper_Phase(+80.68) as a standard signal.

In Figure 9 the integral characteristics *CW_p,P_*(*t_n_*) are presented calculated for frequencies ν = 2.2 THz (a), 2.88 THz (b) for the Thick Bag signal with the Paper_Phase(+80.68) signal as a standard one. Both frequencies in (a), (b) are detected in the frequency ranges ν = [2.0, 2.24] THz (a), ν = [2.84, 3.08] THz (b). That is, spectral features of the standard Paper_Phase(+80.68) signal at ν = 2.16, 2.88 THz are detected at frequencies ν = 2.2, 2.88 THz in the Thick Bag signal. Therefore, paper is found in the Thick Bag sample.

### 5.3. Detecting RDX Absence in the Si-Based Semiconductors

**Figure 10:**
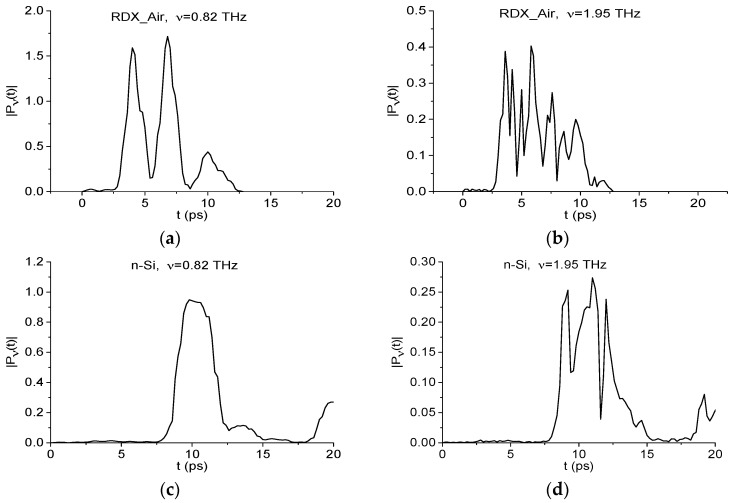
Dynamics of spectral lines of the RDX_Air (**a**,**b**) and n-Si (**c**,**d**) signals at frequencies ν = 0.82 THz (**a**,**c**), 1.95 THz (**b**,**c**).

The n-Si and p-Si spectra in Figure 4a do not contain minima at the frequencies which are equal or close to the main characteristic absorption frequency of the RDX_Air signal at ν = 0.82 THz. The minimum at ν = 1.95 THz is also absent in these spectra, so in order to confirm the absence of RDX in the samples with semiconductors n-Si, p-Si with the help of modified Criteria (6) and (7), we can use the spectral line dynamics of RDX_Air signal at frequencies ν = 0.82, 1.95 THz as standard spectral lines dynamics. In Figure 10 we show the dynamics of spectral lines of the standard RDX_Air signal at frequencies ν = 0.82 (a), 1.95 (b) THz, and in Figure 10c,d—the corresponding dynamics of the n-Si signal.

**Figure 11:**
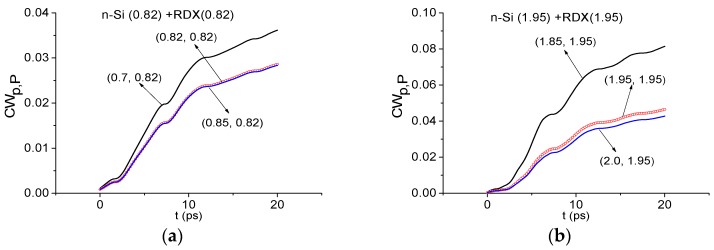
Integral characteristics *CW_p,P_*(*t_n_*) calculated for the frequencies ν = 0.82 THz (**a**); 1.95 THz (**b**) THz for n-Si signal with RDX_Air as a standard one.

Figure 11 shows the integral characteristics *CW_p,P_*(*t_n_*) calculated for the frequencies ν = 0.82 THz (a), 1.95 THz (b) THz for the n-Si signal by using RDX_Air as a standard one. In both cases, these frequencies are not detected—in each frequency range there are characteristics which lie higher than the corresponding values for the standard frequency. We obtain the same result for the p-Si signal.

Integral Criterias (5) and (8) allow one to show the absence of integral correlation even in the case, when the spectrum of the THz signal under investigation contains the minimum at frequency close or equal to characteristic absorption frequency of the standard signal. Therefore, the frequency ν = 2.25 THz is a minimum of the n-Si signal spectrum (Figure 4a), close to absorption frequency ν = 2.2 THz of the RDX_Air (Figure 6).

**Figure 12:**
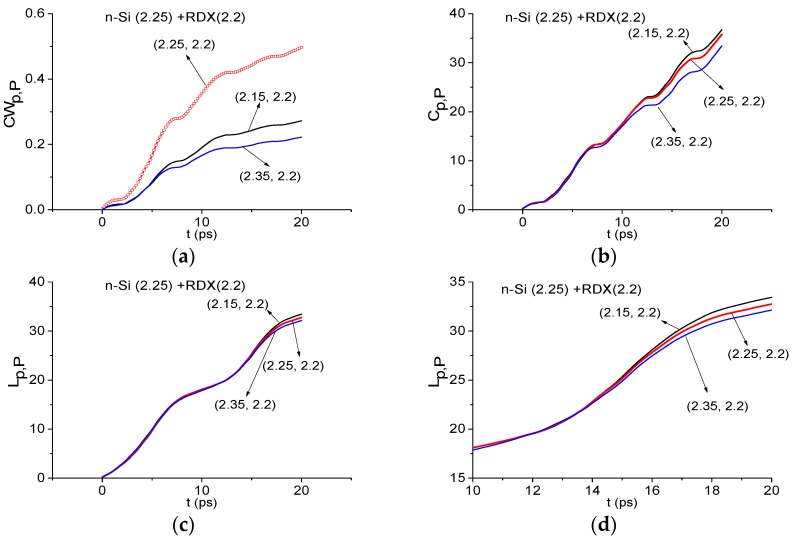
Integral characteristics *CW_p,P_*(*t_n_*) (**a**); *C_p,P_*(*t_n_*) (**b**); *L_p,P_*(*t_n_*) (**c**,**d**) calculated for the frequency ν = 2.25 THz for n-Si signal with RDX_Air as a standard one.

Figure 12a shows the integral correlation characteristic *CW_p,P_*(*t_n_*) calculated for the frequency ν = 2.25 THz. It lies above other characteristics in the frequency range ν = [2.15, 2.35] THz. However, using the integral Criteria *C_p,P_*(*t_n_*)(5) and *L_p,P_*(*t_n_*) (8) one can greatly improve the detection result.

In Figure 12b,c the integral characteristic Equations (5) *C_p,P_*(*t_n_*) and characteristic *L_p,P_*(*t_n_*) (8) do not detect the frequency ν = 2.25 THz in the frequency range ν = [2.15, 2.35] THz. In (d) the characteristic for ν = 2.25 THz is shown in the reduced time interval *t* = [10, 20] ps, where one can see another characteristic lying above it. Therefore, integral Criteria (5) and (8) show that the frequency ν = 2.25 THz is not absorption frequency of RDX in the n-Si signal. The same result is obtained for the p-Si signal. In the same way, it is possible to show the absence of the spectral features of HMX and PETN in the n-Si, p-Si signals.

**Figure 13:**
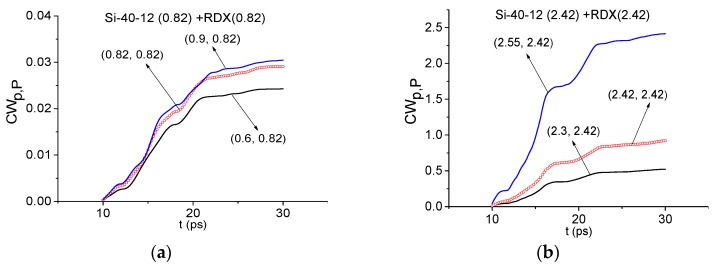
Integral characteristics *CW_p,P_*(*t_n_*) calculated for the frequencies ν = 0.82 THz (**a**); 2.42 THz (**b**) THz for Si-40-12 signal with RDX_Air as a standard one.

In Figure 13 the integral characteristics *CW_p,P_*(*t_n_*) are shown for the frequencies ν = 0.82 THz (a), 2.42 THz (b) THz for the Si-40-12 signal by using RDX_Air as a standard one. In both cases, these frequencies are not detected—in each frequency range there are characteristics which lie higher than the corresponding values for the standard frequency. That is, the explosive RDX is absent in the semiconductor Si-40-12. In the same way, it is possible to show the absence of the explosives HMX, PETN in this sample.

We demonstrated that integral Criteria (5) and (8) allow one to show the absence of RDX spectral features even in the case, when the spectrum of the THz signal under investigation contains a minimum at a frequency close or equal to the characteristic absorption frequency of the standard signal. In order to enhance the detection reliability it is necessary to use different types of integral criteria simultaneously.

## 6. Conclusions

We showed the essential limitations of the standard THz TDS method for the detection and identification of substances, which are based on comparing their stationary spectra. As examples of neutral substances under analysis, we used a thick paper bag and the silicon-based semiconductors n-Si, p-Si and a silicon wafer. We demonstrated that the standard THz TDS method detects the spectral features of the explosives RDX, HMX, PETN and illicit drug MDMA in these samples. At the same time, based only on this method, it is impossible to show the actual absence of hazardous substances in the samples. This fact makes the THz TDS method insufficient for the reliable identification of substances not only under real conditions (at a long distance and high relative humidity), but also under laboratory conditions (at a short distance and low relative humidity). It should be stressed that this also makes it difficult to construct a device that can detect dangerous substances in the real world by means of the standard THz TDS method, as it will lead to a large number of false positives—for example, instead of a cell phone such a device will detect RDX in the pocket of a person.

At the same time, the proposed integral criteria and SDA-method allow us to detect the absence of dangerous substances in the samples under investigation and to find paper in the Thick Paper Bag sample. In order to enhance the detection reliability it is necessary to use the different types of integral criteria simultaneously.

We also showed that the Thick Paper Bag and Paper_phase(+86.68) samples demonstrate spectral features similar to those of pure microcrystalline cellulose, and the silicon-based semiconductor samples from different laboratories (n-Si, p-Si and Si-40-12) demonstrate similar spectral features of pure silicon.

Thus, the discussed method is a promising and competitive one for the effective detection and identification of various substances both under laboratory and real conditions, in comparison with the THz TDS method, based on comparison of the substances’ spectra. The method can be used with success for security problems solving, for non-destructive testing, as well as for quality control in the pharmaceutical and food industry.

## Abbreviations

The following abbreviations are used in this manuscript:

THz TDS	Terahertz time-domain spectroscopy
SDA-method	Spectral Dynamics Analysis method
FDR	Frequency detection range
